# High planting density induces the expression of GA3-oxidase in leaves and GA mediated stem elongation in bioenergy sorghum

**DOI:** 10.1038/s41598-020-79975-8

**Published:** 2021-01-08

**Authors:** Ka Man Jasmine Yu, Brian McKinley, William L. Rooney, John E. Mullet

**Affiliations:** 1 Department of Biochemistry and Biophysics, Texas A&M University, College Station, TX 77843-2128 USA; 2 Department of Soil and Crop Sciences, Texas A&M University, College Station, TX 77843-2128 USA

**Keywords:** Biofuels, Light responses, Plant development, Plant hormones, Plant physiology, Plant signalling

## Abstract

The stems of bioenergy sorghum hybrids at harvest are > 4 m long, contain > 40 internodes and account for ~ 80% of harvested biomass. In this study, bioenergy sorghum hybrids were grown at four planting densities (~ 20,000 to 132,000 plants/ha) under field conditions for 60 days to investigate the impact shading has on stem growth and biomass accumulation. Increased planting density induced a > 2-fold increase in sorghum internode length and a ~ 22% decrease in stem diameter, a typical shade avoidance response. Shade-induced internode elongation was due to an increase in cell length and number of cells spanning the length of internodes. *SbGA3ox2* (Sobic.003G045900), a gene encoding the last step in GA biosynthesis, was expressed ~ 20-fold higher in leaf collar tissue of developing phytomers in plants grown at high vs. low density. Application of GA3 to bioenergy sorghum increased plant height, stem internode length, cell length and the number of cells spanning internodes. Prior research showed that sorghum plants lacking phytochrome B, a key photoreceptor involved in shade signaling, accumulated more GA1 and displayed shade avoidance phenotypes. These results are consistent with the hypothesis that increasing planting density induces expression of *GA3-oxidase* in leaf collar tissue, increasing synthesis of GA that stimulates internode elongation.

## Introduction

Agriculture provides food for direct human consumption, forage and feed for animals, and biomass for production of biofuels and bioproducts. Current projections indicate that agricultural productivity will need to improve by 75–100% by 2050 to meet the needs of the world’s increasing population^1–4^. Increased productivity due to the conversion of additional land for agricultural production, irrigation, high nitrogen fertilizer utilization and genetic gains associated with the ‘green revolution’ are slowing and insufficient to meet projected needs^5–7^. Moreover, increasing temperature and drought associated with climate change are expected to create additional production challenges, especially for grain crops^8^. Moreover, the increases in agricultural productivity need to occur while reducing or eliminating agriculture’s greenhouse gas footprint, currently 23% of total greenhouse gas emissions^9^.

Numerous bioenergy crops are under development including poplar^10,11^, switchgrass^12^, sugarcane^13–15^, Miscanthus^16,17^, and bioenergy sorghum^18,19^. These crops will be grown in different regions of production landscapes that are optimal for forests, perennial grasses, or annual grasses in order to maximize overall productivity, resilience, and sustainability^20^. Bioenergy sorghum hybrids are annual C4 grasses designed for deployment on land environmentally and/or economically marginal for production of most food crops^18,19^. Bioenergy sorghum produces biomass that can be used for forage or converted to biofuels and specialty bioproducts^21,22^. First generation bioenergy sorghum hybrids were ~ 4 m tall with the genetic potential to accumulate ~ 40 Mg of biomass per hectare under good growing conditions^23,24^. Bioenergy sorghum hybrids including sweet sorghum types have good nitrogen use efficiency^25,26^, broad adaptation^27,28^ and high GHG displacement metrics (75% for biomass conversion to bioethanol; 90–95% for bioenergy)^23^.

Stems are the largest sinks for biomass in bioenergy sorghum accounting for ~ 80% of harvested biomass^23^. During bioenergy sorghum development, increases in radiation use efficiency (RUE) were associated with canopy closure and the onset of rapid stem and internode elongation^23^. This suggested that increased sink strength due to stem growth could contribute to biomass yield. Sorghum internode elongation is modulated by *Dw1*, a brassinosteroid signaling protein^29–31^, *Dw2*, an AGCVIII kinase^32^, *Dw3*, an auxin efflux transporter^33,34^ and gibberellin (GA)^35^. Prior research in other plant systems showed that GA biosynthesis and signaling affects stem growth^36^, cell elongation and cell division^37^. GA activates gene expression in part by interacting with and inducing the turnover of DELLAs, repressors of GA modulated gene expression^38^.

The height of bioenergy sorghum plants and the length of stem internodes is increased by shading^39^. Shading causes a reduction in the ratio of R:FR light that is detected by phytochromes, red-light photoreceptors that mediate shade avoidance responses^40–42^. Sorghum genotypes that lack phytochrome B (*phyB-1*) express shade avoidance phenotypes such as reduced tillering, early flowering, increased shoot growth, gibberellin accumulation and increased ethylene biosynthesis^43–48^. Shade induced stem elongation increases canopy height helping plants outcompete neighboring plants for sunlight, reduces branching (tillering), and induces early onset of leaf senescence, flowering and seed dormancy.

The biochemical basis of shade avoidance responses (SAR) and SAR-signaling pathways has been studied extensively^42,49–52^. Phytochromes, cryptochromes, phototropins, and UV-B photoreceptors monitor the light environment and mediate responses to nearby plants (proximity sensing) and canopy shade^53–56^. PHYTOCHROME B (PHYB) plays a key role in SAR-signaling by detecting variation in the ratio of red (R) and far-red (FR) light^57^. Direct sunlight has a high ratio of R:FR, while light within canopies has a lower ratio of R:FR because red light is absorbed by chlorophyll. Photoactivated PHYB (Pfr) enters the nucleus, interacts with PHYTOCHROME INTERACTING FACTORS (PIFs) and mediates their degradation by E3 ligases and 26S proteases^42,58^. PIFs are a family of bHLH transcription factors that act as the primary hub for signaling cascades that regulate cell elongation^59^. PHYB in its inactive Pr state will not enter the nucleus, allowing the accumulation of PIFs, which activate growth-promoting genes that contain E-box and G-box motifs, such as those involved in the biosynthesis and transport of the plant hormones auxin, gibberellins, brassinosteroids, cytokinins and ethylene^60–67^.

Elevated biomass yield of bioenergy sorghum relative to grain sorghum is correlated with longer growing seasons and increased plant height. In addition, bioenergy sorghum is typically grown at higher planting density (~ 132,000 plants/ha)^23^ compared to grain crops such as maize (~ 40 to 80,000 plants/ha)^68^. High planting density increases canopy shading, which is expected to induce bioenergy sorghum stem elongation as well as other shade avoidance responses. In order to better understand the interaction among these factors, bioenergy sorghum was grown in the field at four different planting densities and morphometric and biomass data was collected 60 days after emergence (DAE). The results indicate that high planting density induces changes in stem growth and morphology consistent with shade avoidance responses. The expression of *GA 3-oxidase* (*GA3ox2*) was increased in the leaf base and leaf blade:leaf sheath (LB:LS) collars at high planting density indicating that leaf-derived GA could play a key role in regulating internode elongation in bioenergy sorghum under field conditions.

## Results

### High planting density increases plant height and stem internode elongation

**Figure 1:**
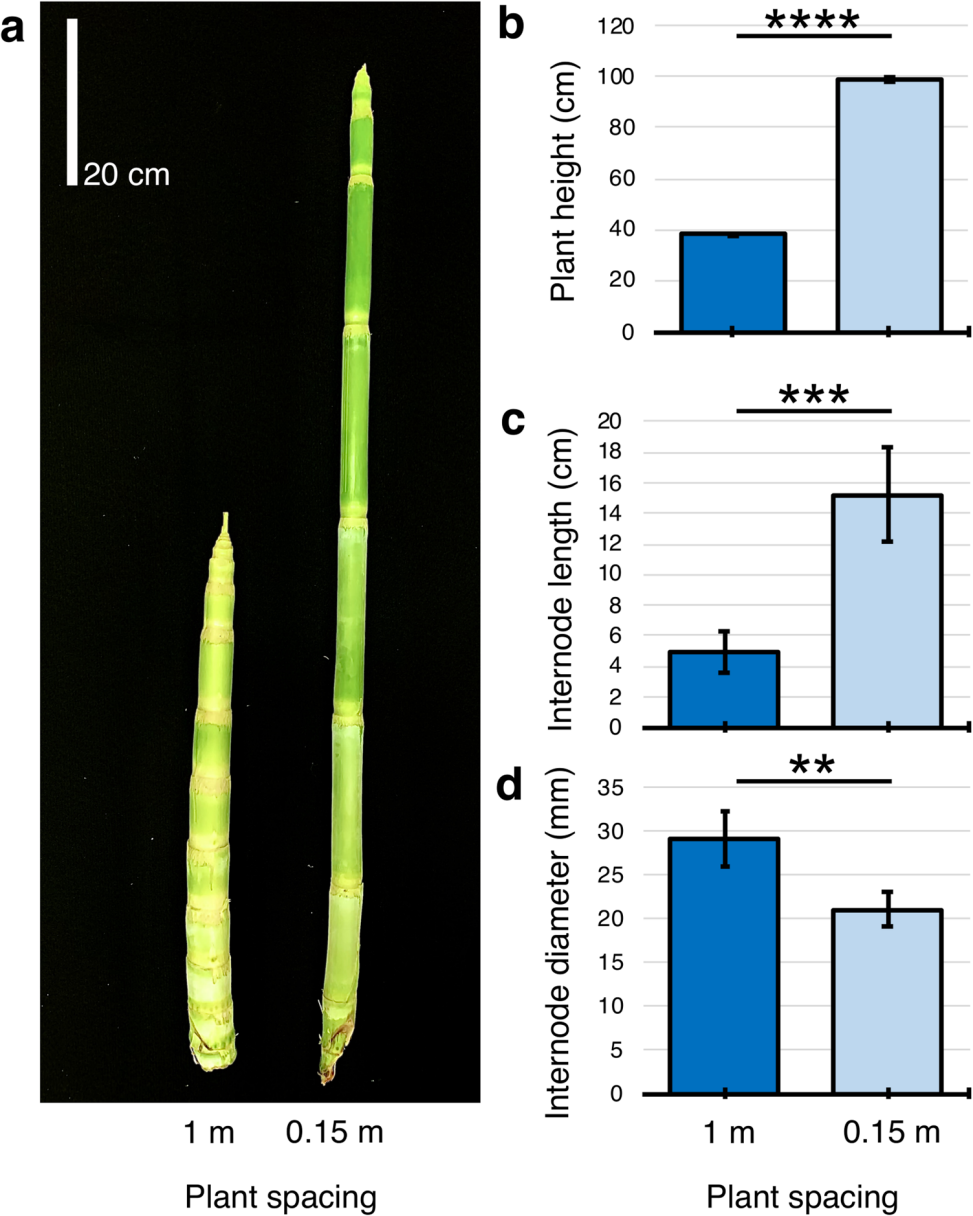
Plant spacing alters sorghum stem growth and morphology. (**a**) Photograph of stems of bioenergy sorghum plants grown for 60 days in the field at 1 m or 0.15 m spacing, (**b**) average height of plants grown at 1 m and 0.15 m spacing. Average length (**c**) and diameter (**d**) of elongated internodes of plants grown at 1 m or 0.15 m spacing. Asterisks indicate two-tailed P value; ****P < 0.0001, ***P = 0.0007, **P = 0.0019, by Welch’s t test (n = 5). Error bars: SEM.

To analyze bioenergy sorghum’s response to shading, the bioenergy sorghum hybrid TX08001 was planted in field plots with 0.76 m row spacing and plants within rows were thinned to a plant spacing of 1 m, 0.5 m, 0.25 m and 0.15 m (~ 20,000 to 132,000 plants/ha). Planting density was maintained for the duration of the experiment by removal of tillers. Plants were harvested at 60 DAE and plant height, stem and internode morphology, and stem and leaf biomass were quantified (Fig. 1, see Supplementary Fig. S1). Plants grown at 0.15 m spacing (0.15 m) were significantly taller and had longer and thinner internodes compared to plants grown at 1 m spacing (1 m) (Fig. 1a–d). However, planting density did not have a significant impact on total plant, leaf or stem dry weight, although the dry weight of stems of plants grown at 0.15 m was somewhat higher compared to plants grown at lower planting densities (see Supplementary Fig. S1).

**Figure 2:**
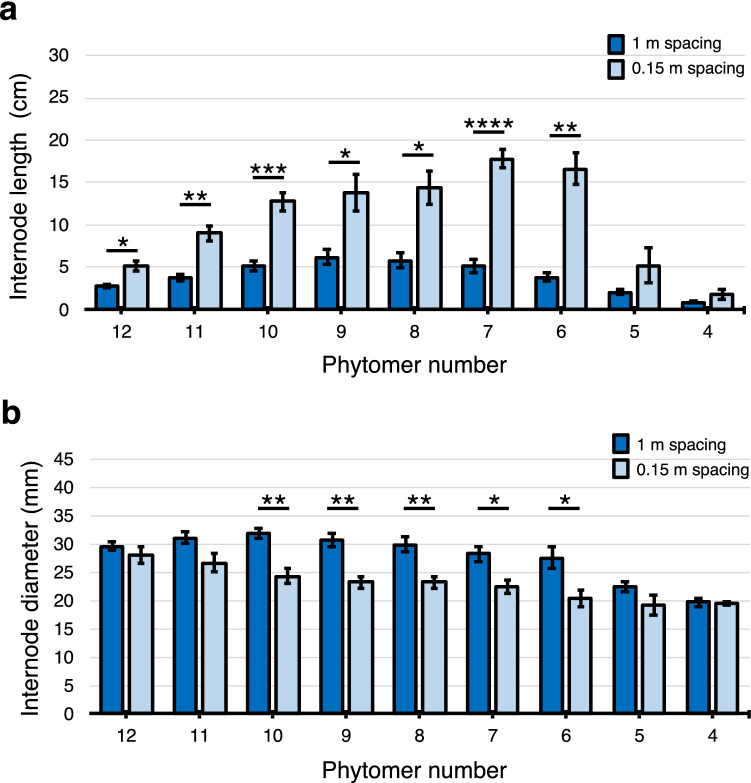
Plant spacing alters sorghum stem internode growth during development. Average internode lengths (cm) (**a**) and diameters (mm) (**b**) of 9 phytomers of bioenergy sorghum grown at 1 m and 0.15 m spacing for 60 days (DAE) in the field. Phytomer 12 is located at the base of the stem. Asterisks indicate two-tail Welch’s t test, ****P < 0.0001, ***P < 0.001, **P < 0.005, *P < 0.05, by one-way ANOVA (n = 5). Error bars: SEM.

The sorghum shoot apical meristem produces a phytomer comprised of a leaf blade, leaf sheath and subtending node-internode approximately every 3–4 days during the adult vegetative stage. In this study, phytomers were numbered sequentially from the youngest, located just below the shoot apical meristem, to the oldest phytomer, located near the base of the shoot. TX08001 plants grown at 1 m and 0.15 m contained ~ 9 visible internodes associated with phytomers 4–12 at 60 DAE (Fig. 2). Phytomers are produced sequentially by the shoot apical meristem during vegetative growth, therefore the youngest visible internode is associated with phytomer 4 located near the top of the plant (Fig. 2a,b). The oldest internode (Internode 12) located near the base of the stem was shorter than most of the internodes above it, except for the youngest non-elongated internodes located close to the apex (Fig. 2a). Fully elongated internodes of plants grown at 0.15 m were longer than the corresponding internodes of plants grown at 1 m (Fig. 2a). The difference in internode length increased as a function of when elongation occurred during plant development. Internode 7 of plants grown at 0.15 m was ~ 3 times longer than internode 7 of plants grown at 1 m. Internode 7 was the longest internode in plants grown at 0.15 m whereas internodes 8/9 were the longest internodes of plants grown at 1 m. Internode diameters showed the opposite response to planting density such that plants grown at high planting density (0.15 m) had smaller internode diameters compared to low planting density (1 m) (Fig. 2b). The diameter of internode 12 at all planting densities was similar, but the diameter of internode 7 of plants grown at 0.15 m was ~ 22% smaller than plants grown at 1 m (Fig. 2b). Overall, the volume of the stems of plants grown with 0.15 m was ~ 39% greater than plants grown at 1 m at 60 DAE (see Supplementary Table S1). The leaves of phytomers 6–8 of plants grown at high vs. low planting density showed relatively small differences in length and width (see Supplementary Table S2). Taken together, bioenergy sorghum responds to higher planting density by 60 DAE, by increasing the length and reducing the diameter of internodes.

### Shade-induced changes in cell length and number per internode

**Figure 3:**
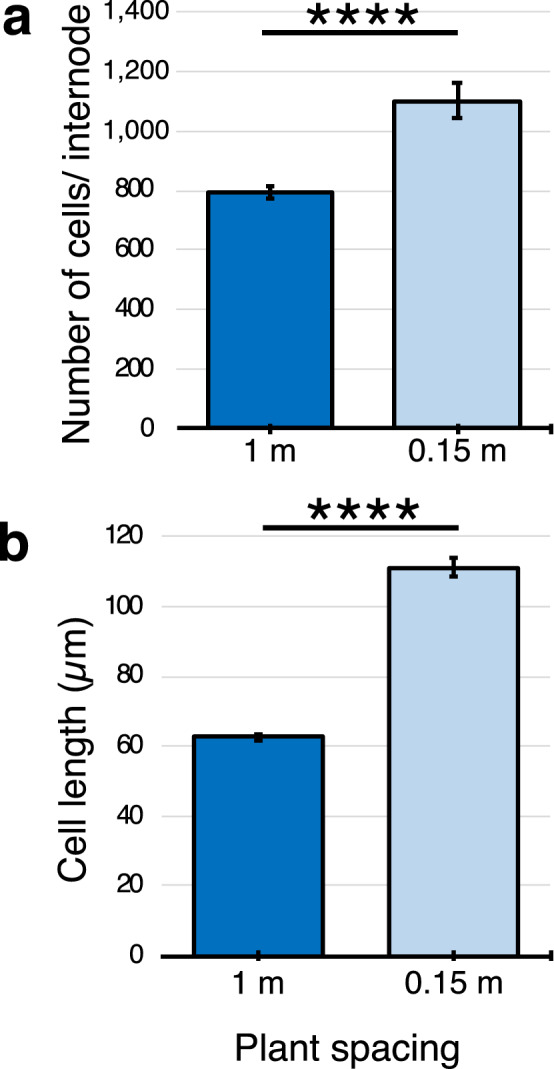
Plant spacing affects the number and length of cells in sorghum internodes. (**a**) The average number of cells spanning fully elongated internodes of phytomer 7 and (**b**) average length of cells in phytomer 7 of plants grown at 1 m and 0.15 m spacing. Asterisks indicate two-tailed P value, ****P < 0.0001, by Welch’s t test [n = 96 (1 m), n = 84 (0.15 m)]. Error bars: SEM.

The differences in length of internodes of plants grown at varying planting densities could be due to variation in the number of cells that span the length of an internode, cell length or both factors. To investigate this question, plants were grown in a greenhouse at 1 m and 0.15 m for 60 days prior to analysis of the number and length of cells in a fully elongated internode. Longitudinal sections that span the length of internode 7 were obtained and cell lengths and numbers were quantified (Fig. 3). The analysis showed that there were ~ 28% more cells across the length of internode 7 in plants growth at high density compared to low density (Fig. 3a). The analysis also showed that cells that comprise internode 7 from plants grown 0.15 m were ~ 44% longer than cells from internode 7 of plants grown at 1 m spacing (Fig. 3b). These results indicate that both cell elongation and cell proliferation contribute to the increase in internode length observed at increased planting density.

### Potential role of GA in shade-induced stem elongation

Phytochrome B (phyB) is a key red light photoreceptor involved in SAR-signaling^45,57^. The sorghum genotype 58 M encodes *phyB-1*, a non-functional version of phyB^45^. When compared to near isogenic lines that encode *PHYB* (i.e., 100 M, 90 M, 80 M, 60 M), 58 M (*phyB-1*) exhibits phenotypes associated with shade avoidance including early flowering, elongated shoots, reduced tillering and narrow leaves^43^. 58 M plants also accumulate 2–6 times higher levels of GA^46^. Moreover, treatment of 60 M or 80 M (*PHYB*) with GA induced the SAR-associated phenotypes observed in 58 M (*phyB-1*)^44^. Since phyB mediates many SAR responses, we hypothesized that increased shading of TX08001 in the field could induce an increase in GA synthesis or signaling that results in increased stem elongation.

**Figure 4:**
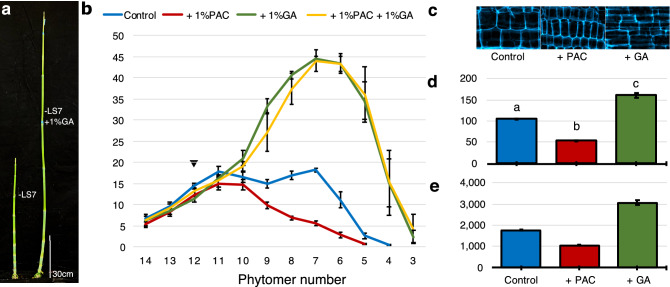
GA3 alters sorghum internode length, cell length and number. (**a**) Photograph of sorghum stems from control plants 14 days after removal of the leaf sheath (LS) from phytomer 7 (− LS) and plants treated with GA3 after LS removal (− LS, 1% GA3) (right). (**b**) Average length of internodes of control (blue line), and plants treated with 1% GA3 (green line), 1% PAC (red line) or both compounds (yellow line). Site of LS removal and GA3/PAC application is marked (solid inverted triangle). (**c**) Micrographs of longitudinal sections from the middle section of fully elongated internode from phytomer 7 (solid star) stained for cellulose. (**d**) Average length of cells and (**e**) average number of cells spanning the length of internode 7 following 14 days of treatment. One-way ANOVA, followed by Tukey comparison test, indicated significant differences between control, + PAC and + GA conditions with P < 0.0001 (n = 291). Different letters indicate significance. Error bars: SEM.

Most of the prior research on phyB signaling and GA biosynthesis in sorghum utilized genotypes that were recessive for the stem dwarfing loci *Dw2* and *Dw3* that reduce internode lengths and most studies were conducted at the seedling stage or post floral initiation^44,46^. Therefore, in the current study, the impact of GA on internode elongation in the bioenergy hybrid R07020 (*Dw1Dw1Dw2Dw2Dw3Dw3*) was examined during the vegetative phase by treating plants with GA3 or the GA biosynthesis inhibitor Paclobutrazol (PAC). GA (or PAC) was added to the lower part of the stem, below phytomers that contain elongating internodes, to see if variation in GA would alter internode growth. This was done by removing the leaf blade (LB) and leaf sheath (LS) of phytomer 7, a phytomer located just below the internode growing zone, and applying GA3 or PAC in lanolin to the excised LS where it joins the stem. Plants were then grown for an additional 14 days before analysis of stem and internode lengths (Fig. 4a,b). Removal of the LS had minimal impact on stem length (see Supplementary Fig. S2), however, addition of 1% GA3 greatly stimulated stem growth (Fig. 4a). Four to five additional phytomers were formed during the GA3 or PAC treatments, therefore at harvest, phytomer 12 corresponds to the phytomer treated with GA3 or PAC at the start of the experiment (Fig. 4b, downward arrow). The length of the internode associated with phytomer 12 was not altered by GA3 or PAC treatment because the internode was fully elongated at the start of the treatment. At the end of the treatment, phytomers 7–10 contained internodes that had reached full elongation during the treatment and phytomers 4–6 contained internodes that were still in various stages of elongation (Fig. 4b). GA3 treatment had minimal impact on the length of the internode in phytomer 11, caused a small increase in the length of internode 10, and had an increasingly large impact on the lengths of internodes 9, 8 and 7 (Fig. 4b). At the start of the GA3 treatment, internode 10 was nearly fully expanded, whereas internode 7 was just beginning to start elongation, explaining why GA3 treatment had a greater impact on the growth of internode 7. In the control, rapid internode growth begins between phytomer 4 and 5, and is completed in phytomer 7, a developmental window spanning approximately 9 days. In GA3 treated plants, internode growth begins between phytomer 3 and 4 and is completed in phytomer 6, also approximately 9 days. This indicates that GA3 treatment increased the extent of internode elongation, but not the duration of the elongation process. The appearance of an additional internode in GA-treated plants could indicate that the rate of phytomer production was increased by GA3 treatment, consistent with prior studies showing that GA3 modifies expression of genes that regulate plastochron/phyllochron^69,70^. Addition of 1% PAC to the node (or to upper leaves by foliar spraying, see Supplementary Fig. S3) reduced internode lengths in phytomers 5–9 and reduced the number of visible internodes above the site of application by one (Fig. 4b). The number and length of cells located in internodes of phytomer 7 were measured following removal of the leaf sheath (Control), treatment with PAC (+PAC) or GA3 (+GA) (Fig. 4c–e). The length of internode cells was increased by GA3 treatment and decreased by PAC (Fig. 4d). The number of cells spanning the length of the internode was also increased by treatment with GA, compared to control and treatment with PAC. These results indicate that modification of GA levels can alter the length of bioenergy sorghum internodes by altering cell lengths and the number of cells spanning internodes, similar to shading.

### Shade-induced expression of *GA3-oxidase*

Shade-induced internode elongation could be due to an increase in GA biosynthesis, GA-signaling, and/or other factors. Variation in GA biosynthesis is often correlated with the expression of genes encoding *GA3ox* the final step in the GA biosynthetic pathway^64,69^. Two sorghum genes annotated as encoding *GA3ox* have been identified; Sobic.009G064700 (*SbGA3ox1*), a homolog of *OsGA3ox1,* and Sobic.003G045900 (*SbGA3ox2*), a homolog of *OsGA3ox2* and *ZmGA3ox2*^69,71^. In maize, *ZmGA3ox1* is expressed at very low levels except in tassels^71^. Analysis of RNA-seq data from BTx623 tissues^72^ showed that *SbGA3ox1* was expressed at very low levels in all tissues and developmental stages represented in the sorghum transcriptome compendium including stems (see Supplementary Table S3). In rice, *OsGA3ox2* is more highly expressed in young leaves^69^ and in maize, *ZmGA3ox2* is expressed at low and varying levels in several organs and tissues^71^. *SbGA3ox2* was expressed at relatively low levels in tissues of the sorghum transcriptome compendium except dry and germinating seeds where expression was elevated (see Supplementary Table S3).

**Figure 5:**
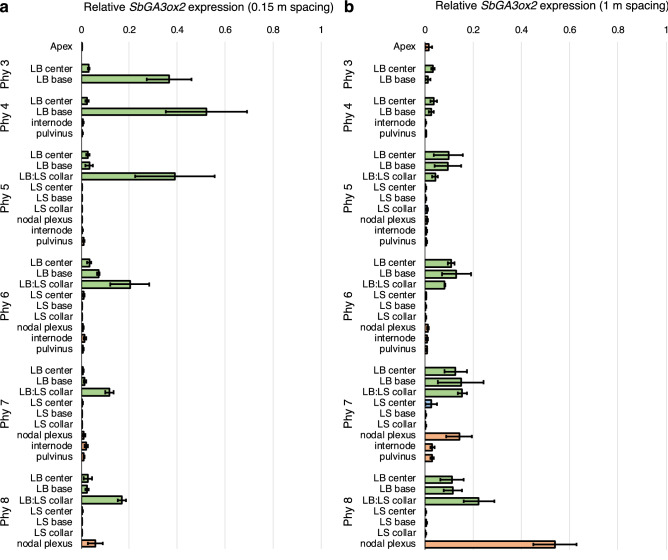
Relative expression of *SbGA3ox2* in sorghum grown at 0.15 m (**a**) and 1 m (**b**) spacing. Tissues were collected from the shoot apex (Apex), mid leaf blade (LB center), base of the leaf blade (LB base), tissue between the leaf blade and leaf sheath (LB:LS collar), mid leaf sheath (LS center), base of the leaf sheath (LS base), leaf sheath collar (LS collar), stem nodal plexus, internode, and pulvinus of phytomers (Phy) 3–8. Relative expression is shown in bar graphs (leaf blade = green, leaf sheath = blue, stem = brown). Expression values are the average of three biological replicates. Error bars: SEM. Photograph and diagram of the development of sorghum phytomer tissues is shown in Supplementary Figure S4.

The sorghum transcriptome compendium contains RNA from vegetative and reproductive tissues, but the compendium does not contain RNA-seq profiles comparing shaded to non-shaded plants. Therefore, qRT-PCR was used to conduct a systematic analysis of *SbGA3ox2* expression in the shoot apex, leaf blade (LB), leaf sheath (LS), and stem tissues that comprise phytomer 3 (prior to internode elongation) through phytomer 8 (fully elongated internodes) of vegetative plants grown at high and low planting density. Leaf tissue samples were collected from the mid-point of the leaf blade (LB center), the growing zone located at the base of the leaf blade (LB base), and the LB:LS collar tissue (see Supplementary Fig. S4). Tissues were also collected from the middle of the leaf sheath (LS center), leaf sheath base growing zone (LS base), LS: stem collar tissue (LS collar) (see Supplementary Fig. S4). Stem tissues collected included the nodal plexus, a stem nodal tissue where the leaf sheath joins the stem, internodes, and the pulvinus, a tissue located between the internode growing zone and the nodal plane^73^ (see Supplementary Fig. S4). Relative expression of *SbGA3ox2* in these tissues was quantified using qRT-PCR (Fig. 5). At 0.15 m spacing (high planting density), *SbGA3ox2* expression was highest in the LB base and/or LB:LS collar of each phytomer, followed by tissues in the middle of the leaf blade (Fig. 5a, LB center). *SbGA3ox2* expression in the LB:LS collar was higher in phytomers 3–5 that contain elongating internodes compared to phytomers 7–8 that contain fully elongated internodes. Plants growing at 1 m spacing (low planting density) showed expression of *SbGA3ox2* in the LB center, LB base, and LB:LS collar tissues, with somewhat higher expression in phytomers 5–8 (Fig. 5b). In addition, expression of *SbGA3ox2* was low in stems of phytomers 3–6, however expression increased in the stem nodal plexus of phytomers 7–8 that contain fully elongated internodes.

**Figure 6:**
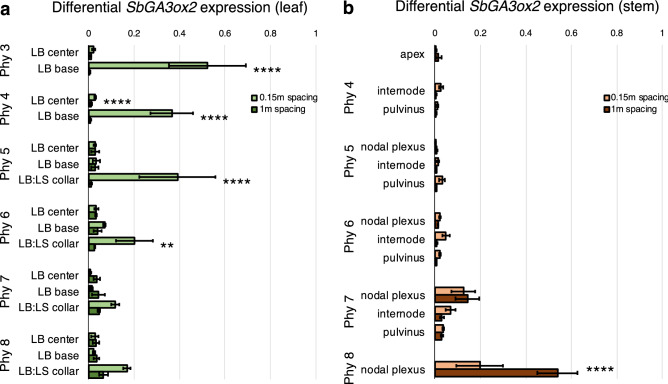
*GA3ox2* is differentially expressed at high vs. low plant spacing. (**a**) Differential expression of *SbGA3ox2* in the leaf blade (LB) and leaf blade/leaf sheath collar tissue (LB:LS collar) of phytomers 3–8 of plants grown at 0.15 m spacing (high density) and 1 m spacing (low density). (**b**) Differential expression of *SbGA3ox2* in the stem apex, nodal plexus, internode and pulvinus of plants grown at 0.15 m and 1 m spacing. Expression values are the average of three biological replicates. Differences in expression were analyzed by one-way ANOVA followed by Tukey comparison test. ****P < 0.0001, **P < 0.005. Error bars: SEM.

Differential expression of *SbGA3ox2* in leaf and stem tissues of plants grown at high planting density vs. low planting density is shown in Fig. 6. This analysis showed that *SbGA3ox2* is expressed at much higher levels in the LB base of phytomers 3–4 and LB:LS collar tissues of phytomers 5–6 in plants grown at high planting density compared to low planting density (Fig. 6a). In contrast, expression of *SbGA3ox2* in stem nodal plexus tissue of phytomers 7–8 was not higher in plants grown at high density compared to low density (Fig. 6b). *SbGA20ox1* expression was also higher in the LB:LS collar and nodal plexus of phytomer 5 in plants grown at high planting vs. low planting density whereas minimal differences in expression were observed in the LB, LS and stem internode (Supplemental Figure S5).

## Discussion

The stems of bioenergy sorghum at the end of the growing season are typically > 4 m long, comprised of > 40 internodes, and account for ~ 80% of harvested biomass^23^. Long stems are correlated with high biomass yield, however, tall plant stature and long thin internodes can also increase susceptibility to lodging^74–76^. Adaptation of maize hybrids to increased planting densities (from 30,000 to 80,000 plants/ha) was a major contributor to increased grain yield per hectare^68^. Bioenergy sorghum is grown at planting densities (~ 132,000 plants/ha) that are higher than optimal for corn^68^. Adaptation of bioenergy sorghum to high planting densities may be needed to optimize biomass yield, composition and standability. Therefore, the current study focused on understanding how variation in planting density affects stem growth and morphology.

The results showed that increasing planting density from ~ 20,000 to ~ 132,000 plants/ha in the field resulted in a ~ 2 to 3-fold increase in stem internode length, a ~ 22% decrease in stem internode diameter, and an overall increase in stem volume of ~ 39% by 60 DAE. The greater length of internodes in plants grown at high density could be accounted for by increases in cell length and the number of cells that span the length of internodes. The planting density induced change in internode morphology occurred without a large impact on total plant biomass accumulation or biomass allocation between leaves and stems, indicating that this initial response to shading primarily alters the morphology of the stem, elevates the canopy and creates greater spacing between leaves. Canopy closure at the highest planting density used in this and previous studies occurs between 60 and 90 DAE therefore proximity sensing of reflected FR light and partial direct shading are probably mediating the observed SAR-induced internode elongation. Increased stem volume at high planting density could benefit bioenergy sorghums that accumulate high levels of stem sucrose (~ 0.5 M)^77^. In addition, plant height and biomass accumulation in bioenergy sorghum panels are correlated when measured after the juvenile phase when stem elongation is rapid^78^. This predicts that longer stems in all types of bioenergy sorghum could potentially improve biomass yield over the course of the growing season even though the initial increase in stem elongation observed here had only limited impact on biomass accumulation. On the other hand, larger stems increase respiratory load and longer and thinner stems could increase the propensity for lodging later in the season. Therefore, identifying and deploying an optimal stem size and morphology is an important long-term goal that could be aided by an understanding of the molecular mechanism that regulates elongation in response to shading.

PHYB is a red light photoreceptor that plays an important role in shade avoidance signaling^53–56^. The sorghum genotype 58 M (*phyB-1*) lacks phytochrome B, exhibits numerous shade avoidance phenotypes and accumulates higher levels of GA1 in leaves compared to near isogenic genotypes that express phyB^44–46^. Treatment of sorghum genotypes, that encode phyB, with GA induced the shade avoidance phenotypes observed in 58 M^44^. This led previous investigators to propose that reduced phyB signaling associated with shading causes an increase in GA that contributes to the observed SAR-phenotypes^43^. The previous studies also showed that GA1 is the predominant GA that accumulates in sorghum, but the investigators were not able to determine how shade and/or phyB signaling alters GA levels.

Since GA3 oxidase is the last step in the GA biosynthetic pathway leading to the formation of GA1 and GA3^79^ and *GA3ox* expression is often correlated with GA biosynthesis and accumulation, we investigated how variation in planting density affects the expression of sorghum genes that encode GA3-oxidase in bioenergy sorghum. Rice, maize and sorghum encode two genes for GA3ox^69,71^. In rice and maize, *GA3ox2* is expressed at low levels in most tissues, with higher expression in young elongating leaves of rice^69^. In growing maize leaves, expression of *ZmGA3ox2* is correlated with GA1 accumulation in the leaf base although other factors such as *GA2-oxidase* also shape the distribution of GA1 across the growing zone^80^. In maize, mutation of *ZmGA3ox2* reduced GA1 levels and caused stem dwarfing^71^. Ectopic overexpression of *GA20-OXIDASE1 (GA20-OX1*) in maize increased GA levels and produced plants with longer but thinner stems similar to sorghum grown at high vs. low density^81^. This indicates that variation in expression of *GA20-OX1* could also cause changes in GA levels during development or in response to environmental variation that alters C4 grass morphology and biomass composition^81^.

In the current study, qRT-PCR was used to characterize *SbGA3ox2* expression in leaf and stem tissues collected from bioenergy sorghum plants growing at low and high planting densities. Sorghum *GA3ox2* expression was quantified in leaf tissues derived from the LB center, LB base (leaf growing zone in phytomers 3–4), and LB:LS collar tissue that spans the ligule in sorghum and maize^82^. It should be noted that delineation of the LB base and LB:LS collar tissue in phytomers 3–4 was not possible, therefore in these phytomers both tissues were combined in the leaf base sample (LB-base/collar tissue). The analysis showed that in plants growing at low density, expression of *SbGA3ox2* was higher in the LB center, LB base and LB:LS collar tissues compared to the shoot apex and stem tissues of most phytomers. Expression of *SbGA3ox2* in leaf tissues increased during development from a low level in phytomers 3–4 that contain elongating leaves and short internodes to higher levels in leaves of phytomers 5–8 which contain full length leaves, elongating internodes (phytomers 5–7) and elongated internodes (phytomer 8). Elevated *SbGA3ox2* expression in leaves was also observed in plants grown at high planting density although expression was higher in leaves of phytomers 3–5 compared to phytomers 7–8. More significantly, *SbGA3ox2* expression was > 20-fold higher in the LB-base/collar tissue of phytomers 3–4 and LB:LS collar tissue of phytomers 4–8 in plants grown at high density compared to plants grown at low density. Increased expression of *SbGA3ox2* in LB-base/collar tissue and LB:LS collar tissue of phytomers 3–5 in plants growing at high planting density is correlated with earlier onset of internode elongation and a ~ 2 to 3-fold increase in internode elongation compared to low planting density.

The results are consistent with the hypothesis that shading increases expression of *SbGA3ox2* in LB:LS collar tissue and this results in increased production of GA that moves to the stem where it stimulates internode growth. LB:LS collar derived GA could move from the LB:LS collar through the leaf sheath, most likely via vascular bundles, and enter the stem at the nodal plexus. GA entering the nodal plexus could move downwards to the internode zone of elongation in the same phytomer and/or upwards into and through the intercalary meristem of the phytomer above the nodal plexus. Application of GA to the nodal plexus (or excised LB:LS collars) stimulated internode elongation by increasing cell length and cell proliferation similar to shade-induced internode elongation. This hypothesis is consistent with studies showing that tobacco leaves are an important source of GA for stem growth^83^ and that mutation of *ZmGA3ox2* causes stem dwarfing in maize^71^. While GA plays a role in shade-induced stem elongation, it is likely that other hormones such as auxin and brassinosteroids are also involved in this response, as in other plants^42,55,56,60,84,85^.

*SbGA3ox2* expression also increased later in development in the nodal plexus of phytomer 8 although expression was not induced by higher planting density. Phytomer 8 and older phytomers that contain fully elongated internodes continue to express genes involved in secondary cell wall formation^77^. In tobacco, it has been shown that leaves are sources of GA for stem secondary growth and fiber differentiation^83^. While grasses lack secondary growth characteristic of trees, increased expression of *SbGA3ox2* in the nodal plexus of older internodes may influence secondary cell wall formation that increases the strength and biomass of older internodes.

Additional research will be needed to determine if phyB-signaling modifies *SbGA3ox2* expression and GA levels in tissues of plants exposed to variation in shading. It will also be important to characterize the cell specific localization of *SbGA3ox2* in the LB:LS collar and to further analyze GA transport from the LB:LS collar to the stem and within the stem. Recent research has identified mechanisms involved in active GA transport^86^ and showed that GA is transported in the endodermis of the root^87,88^. Transport of GA from the LB:LS collar to growing internodes could occur through the endodermis of LS and stem vascular bundles^87,89^. In addition, GA distribution in organs and tissues is shaped by enzymes such as GA 2-oxidases that mediate GA turnover and GA 20-oxidases that provide precursors for GA 3-oxidases^90^. *GA 2-oxidase* expression has been documented in the preligule tissue of maize^91^ and at the transition zone of cell elongation in maize leaf blades^80^. In sorghum, several *GA 2-oxidases* are induced in internodes that are exiting the zone of internode elongation suggesting depletion of GA by these enzymes may help regulate the developmental progression of internode elongation^92^.

## Methods

### Plant growth and field conditions

The bioenergy sorghum hybrid TX08001 was planted in a 16 row × 100 m plot in the PIVET field site at the Texas A&M University Field Station in College Station, Texas (30° 37′ 40″ N, 96° 20′ 3″ W, 100 m above sea level). Plots were planted on May 2, 2017 and emerged on May 9, 2017. Plants with a row spacing of 0.76 m within 4 blocks (16 rows × 10 m) were thinned to 4 different planting densities with 1 m, 0.5 m, 0.25 m and 0.15 m spacing between each plant.

To reduce border effects, all plants were harvested from inner rows of a planting block. Five plants were selected from random locations in each planting density block 60 DAE. Plants were harvested, measured, imaged and weighed. Measurements of individual internode lengths, leaf width and leaf length were obtained using measuring tape. Internode diameters were measured using a Carbon Fiber Composites Digital Caliper, to the nearest millimeter. Harvested stem images were acquired using a 12-megapixel iSight camera. Internode, leaf and leaf sheath and root were weighed for fresh weight (FW) and then bagged individually, dried in an oven at 70 °C for three days before collecting dry weight (DW) data. The calculation of total dry weight per square meter of land was based on plant spacing within plots.

For GA3 and PAC treatments, R07002 plants were grown in a greenhouse under 14-h long days in 5 gallon SmartPots (High Caliper Growing) with Oldcastle Jolly Gardener C/25 Growing Mix (Oldcastle Lawn and Garden) fertilized every 60 days with 1 tbsp Osmocote 14–14-14. Plants were thinned to one plant per pot and grown at 0.15 m spacing.

For microscopic imaging, TX08001 plants were grown in a greenhouse under 14-h long days in 5 gallon SmartPots (High Caliper Growing) with Oldcastle Jolly Gardener C/25 Growing Mix (Oldcastle Lawn and Garden) fertilized every 60 days with 1 tbsp Osmocote 14-14-14. Plants were thinned to one plant per pot and grown at 0.15 m spacing or 1 m spacing.

Seeds were obtained from the Texas A&M Sorghum Breeding Program (College Station, TX).

### GA and PAC treatments

R07020 plants were treated with 1% GA3 (MW = 346.4 g/mol) (SIGMA-ALDRICH G7645-1G), 1% PAC (MW = 293.7 g/mol) or an equal mixture of GA3 and PAC in lanolin at 60 DAE. The plants were treated with the lanolin mixtures at the stem nodal plexus of phytomer 7, after removing the leaf sheath and leaf blade of phytomer 7. The 1% GA3 mixture was made by dissolving 0.93 g of GA3 into 1 mL of 100% ethanol and performing a dilution (100 μL of dissolved into 900 μL of ethanol) to achieve a concentration of 0.093 g/mL. Then, 100 μL of the 0.093 g/mL dissolved GA3 was added to 1 mL of melted lanolin in a hot water bath for a final concentration of 0.0093 g/mL (~ 0.027 M). The mixture was then inverted multiple times to ensure the contents were mixed before the lanolin solidified. The same procedure was use to prepare PAC dissolved in DMSO, for a final concentration of 0.0093 g/mL (~ 0.032 M). The GA3 and PAC mixture was created by mixing 0.93 g each of GA3 and PAC into 2 mL of 100% ethanol and then following the above procedure.

Four biological replicates of R07020 were treated with the PAC foliar spray outside of the greenhouse, and were brought back into the greenhouse after 1 h. 1% PAC foliar spray was made by dissolving 0.78 g of PAC into 1 ml of DMSO and that was subsequently mixed with 9 mL of RO water. 10 mL of the PAC mixture was sprayed onto the canopy of each replicate. 4 controls were treated with a 10 mL mixture of 1 mL DMSO and 9 mL RO water.

### RNA isolation, cDNA sequencing and qRT-PCR

TX08001 was planted at 1 m and 0.15 m densities in the field in May 2019 and tissue collected for gene expression analysis. At 60 DAE, phytomer tissue samples were collected from three biological replicates at each planting density. Tissue was collected from the apex and eight phytomers. Tissue sections were taken from the leaf blade and leaf sheath (center, above collar and collar regions), as well as the stem (nodal plexus, 1 cm internode sections, and pulvinus). Total RNA was extracted from all samples using the Direct-zol™ RNA Miniprep Kit (Zymo Research), and cDNA was synthesized using SuperScript™ III First-Strand Synthesis SuperMix for qRT-PCR (Thermo Fisher Scientific, Invitrogen). The expression of *SbGA3ox2* (Sobic.003G045900) and *SbGA20ox1* (Sobic.001G005300.1) was analyzed using qRT-PCR, using PowerUP™ SYBR™ Green Master Mix (Thermo Fisher Scientific, Applied Biosystems).

The qRT-PCR methods used is described in Casto et al.^93^. For all qRT-PCR experiments, relative expression was determined using the comparative cycle threshold (C_t_) method. Raw C_t_ values for each sample were normalized to C_t_ values of the reference gene *SbUBC* (Sobic.001G526600). Then, ${ddC}_{t}$ values were calculated relative to the sample with the highest expression (lowest C_t_ value). Relative expression values were calculated with the 2^−ddCt^ method^94^. Fold change in gene expression was calculated based on dC_t_, values between the samples with the lowest and highest expression according to the equation FC^2dCt(max)−dCt(min)^. Primer specificity was tested by dissociation curve analysis.

### Microscopy

For microscopic imaging, longitudinal and horizontal hand sections were made using representative TX08001 internodes from fully elongated internodes (phytomer 7) from 0.15 m and 1 m densities, to investigate differences in cell number and length. Longitudinal and horizontal hand sections were also made of using three R07020 internodes from fully elongated internodes (phytomer 7) 14 days after GA3 and PAC treatments.

For cell number and length observation, the sections were stained with a 5% Calcofluor-white solution, a fluorescent blue dye that binds to cellulose, for 1 min and imaged under DAPI fluorescence filter using Carl Zeiss Axio Imager M2, coupled with Axiocam 503, under 5× magnification. ImageJ was used to reduce background and measure cell lengths. Number of cells were calculated by dividing the internode length by cell length.

### Statistical analysis

All statistical analyses (Welch’s t test and one-way ANOVA followed by Tukey comparison test) were performed using GraphPad Prism version 8.4.2 for macOS, GraphPad Software, San Diego, CA, USA, www.graphpad.com.

## Supplementary Information

Supplementary Information
