# Supplementary material for: High planting density induces the expression of GA3-oxidase in leaves and GA mediated stem elongation in bioenergy sorghum
Source: Sci Rep. 2021 Jan 8;11:46. doi: 10.1038/s41598-020-79975-8 (PMC7794234; doi:10.1038/s41598-020-79975-8)
Supplement: Supplementary file 1 — Supplementary Information [file 41598_2020_79975_MOESM1_ESM.pdf]

Supplementary Information

Title: High planting density induces the expression of GA3-oxidase in leaves and GA mediated stem elongation in bioenergy sorghum

Authors: Ka Man Jasmine Yu, Brian McKinley, William Rooney, and John E. Mullet

Supplemental Figures: S1 to S5
Supplemental Tables: S1 to S3

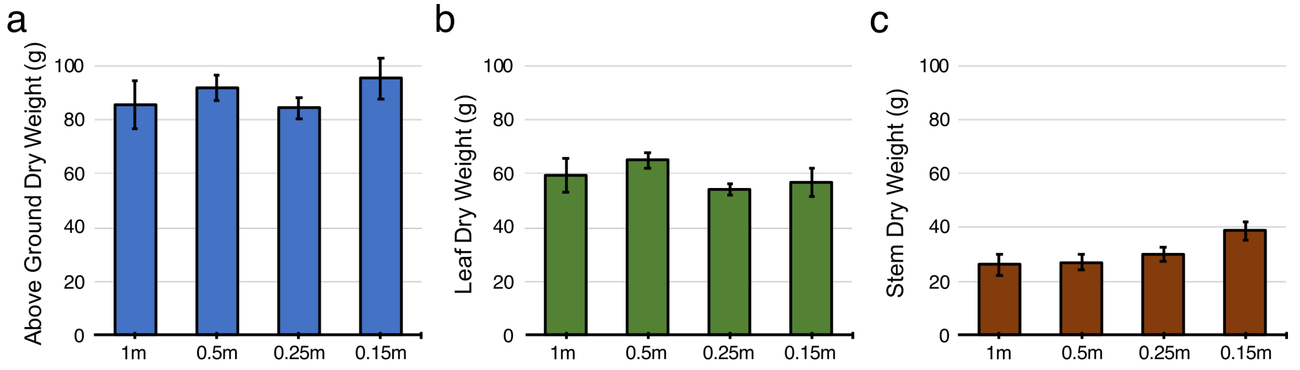

60 DAE	1 m	SE	0.5 m	SE	0.25 m	SE	0.15 m	SE
Leaf DW (LB+LS)	59.41	6.04	64.98	3.03	54.30	2.17	56.75	5.11
Stem DW	26.10	3.79	27.01	2.72	30.20	2.64	38.65	3.26
Above Ground DW	85.51	8.97	91.99	4.83	84.50	3.91	95.40	7.71

Supplementary Figure S1. Shoot dry weight (A), leaf dry weight (B) and internode dry weight (C) of plants grown at 1 m, 0.5 m, 0.25 m and 0.15 m spacing. Error bars represent SEM (n=5 biological replicates per density). One-way ANOVA indicated no statistically significant difference between densities for above ground dry weight: [F(3, 16)=0.61, p=0.62] leaf dry weight: [F(3, 16)=1.09, p= 0.37], or internode dry weight: [F(3, 16)=3.31, p=0.05].

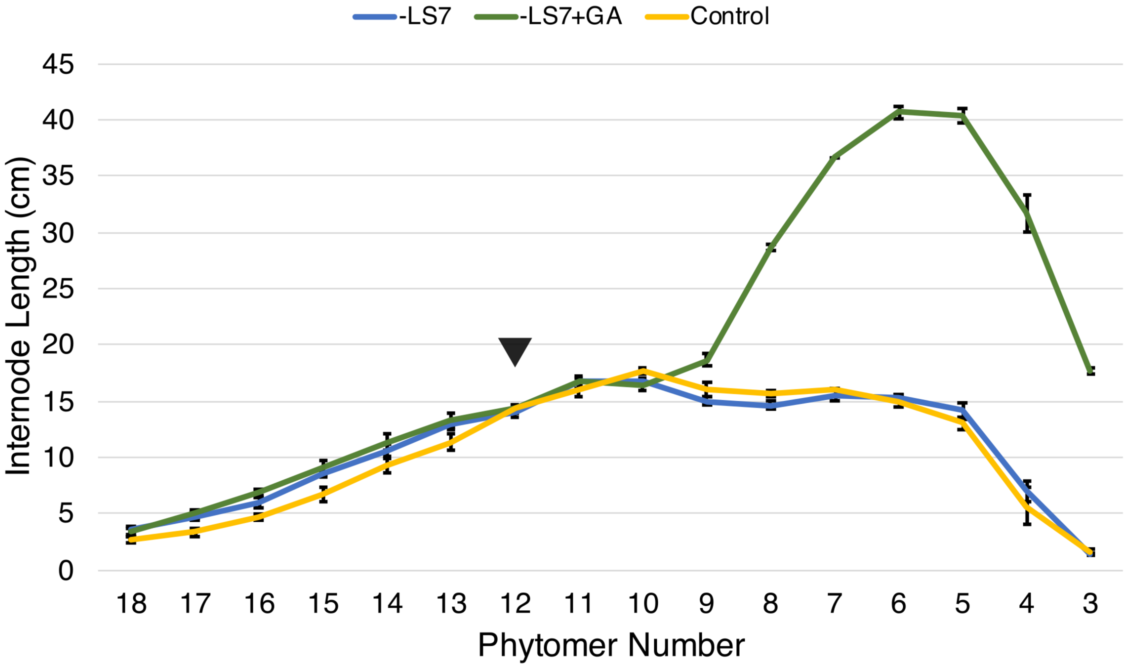

Supplementary Figure S2. Impact of removal of the leaf blade and leaf sheath (-LS) of phytomer 7 and addition of GA (-LS+GA) on the length of internodes 14 days post treatment. ▼ marks LS7 removal.

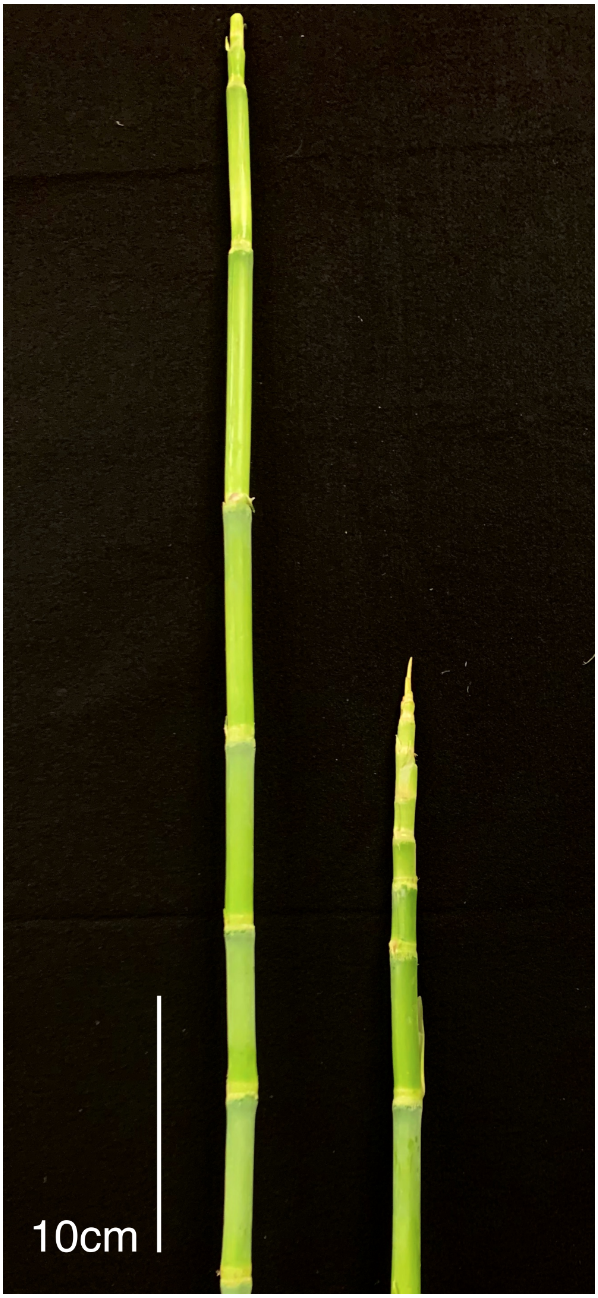

Supplementary Figure S3. R07020 14 days after 1% PAC foliar spray treatment. Left: control. Right: treatment. The most mature (lowest) internode shown in the photo, was the most recent fully elongated internode, prior to 1% PAC foliar spray treatment.

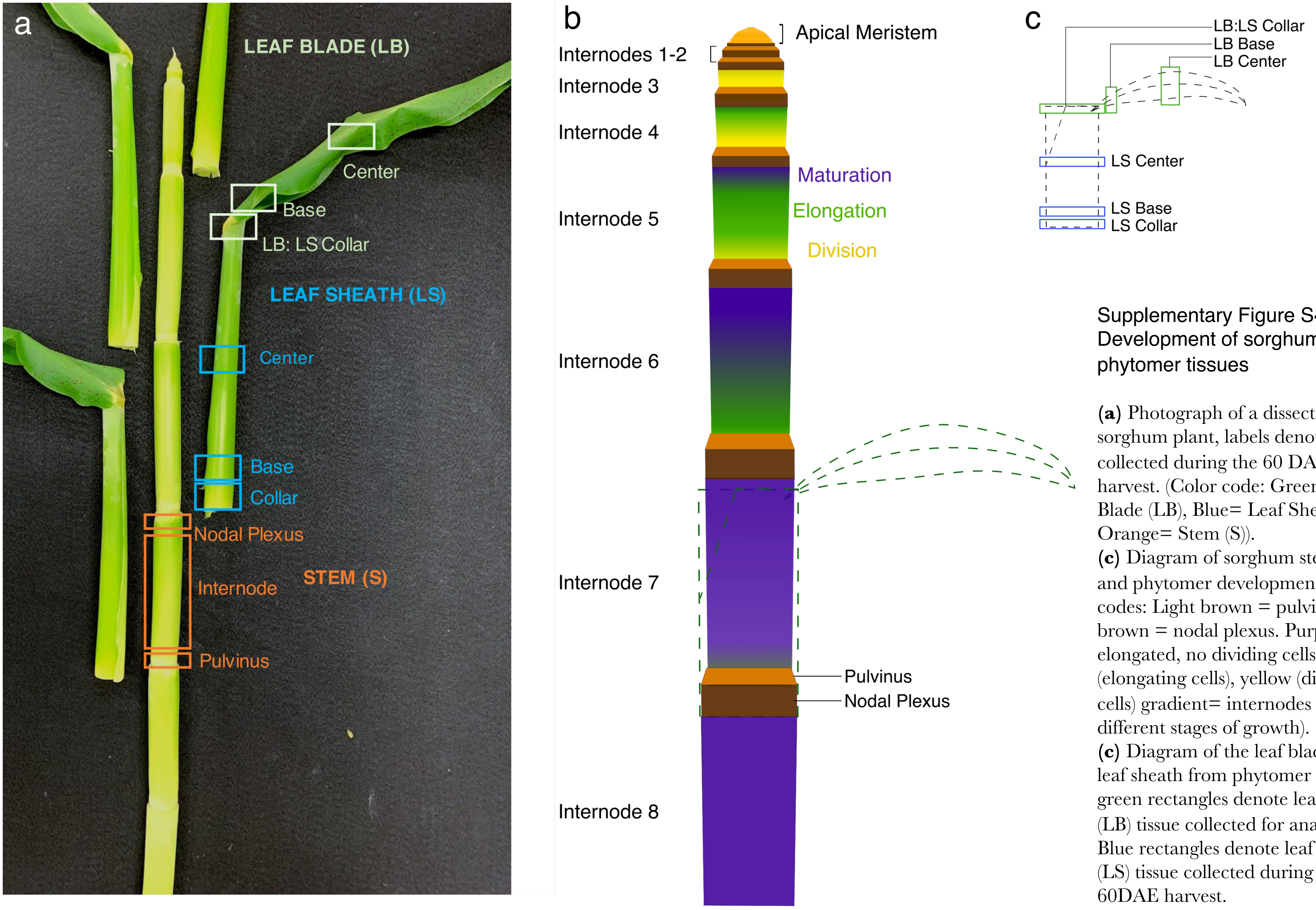

Supplementary Figure S4. Development of sorghum phytomer tissues

(**a**) Photograph of a dissected sorghum plant, labels denote tissue collected during the 60 DAE harvest. (Color code: Green= Leaf Blade (LB), Blue= Leaf Sheath (LS), Orange= Stem (S)).
(**c**) Diagram of sorghum stem tissues and phytomer development. (Color codes: Light brown = pulvinus, dark brown = nodal plexus. Purple (fully elongated, no dividing cells), green (elongating cells), yellow (dividing cells) gradient= internodes at different stages of growth).
(**c**) Diagram of the leaf blade and leaf sheath from phytomer 8. The green rectangles denote leaf blade (LB) tissue collected for analysis. Blue rectangles denote leaf sheath (LS) tissue collected during the 60DAE harvest.

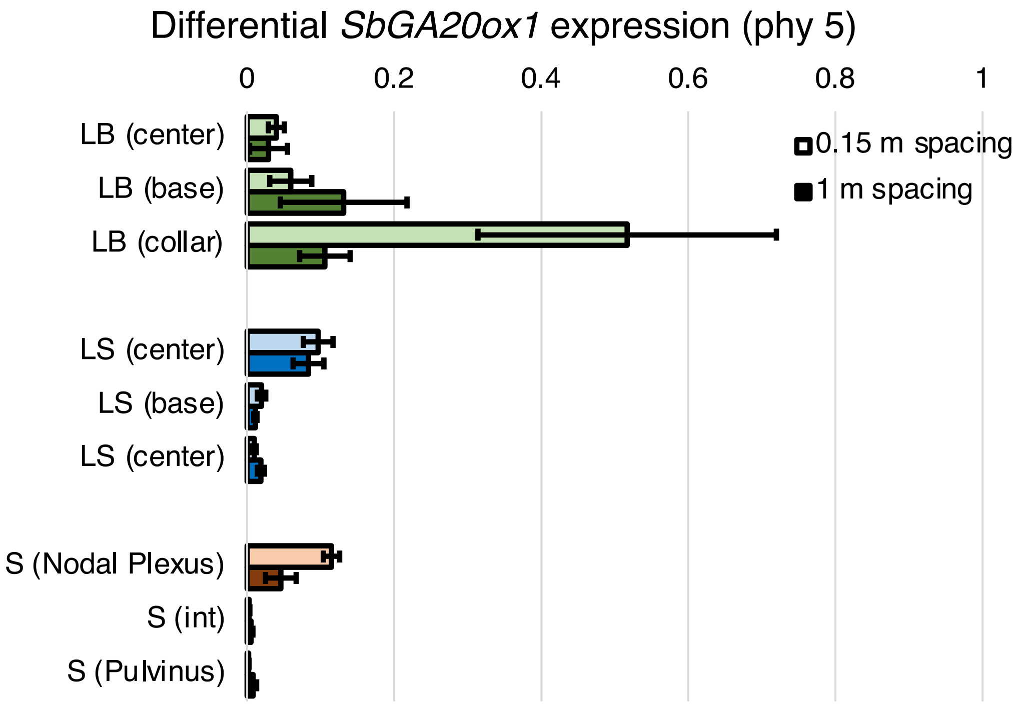

Supplementary Figure S5. *SbGA20ox1* is differentially expressed at high vs. low planting density. Differential expression of SbGA20ox in the leaf blade (LB), leaf sheath (LS) and stem (S) of phytomer 5 of plants grown at 0.15m and 1m spacing. Relative expression is shown in bar graphs (leaf blade = green, leaf sheath = blue, stem = brown). Expression values are the average of three biological replicates. Error bars: SEM.

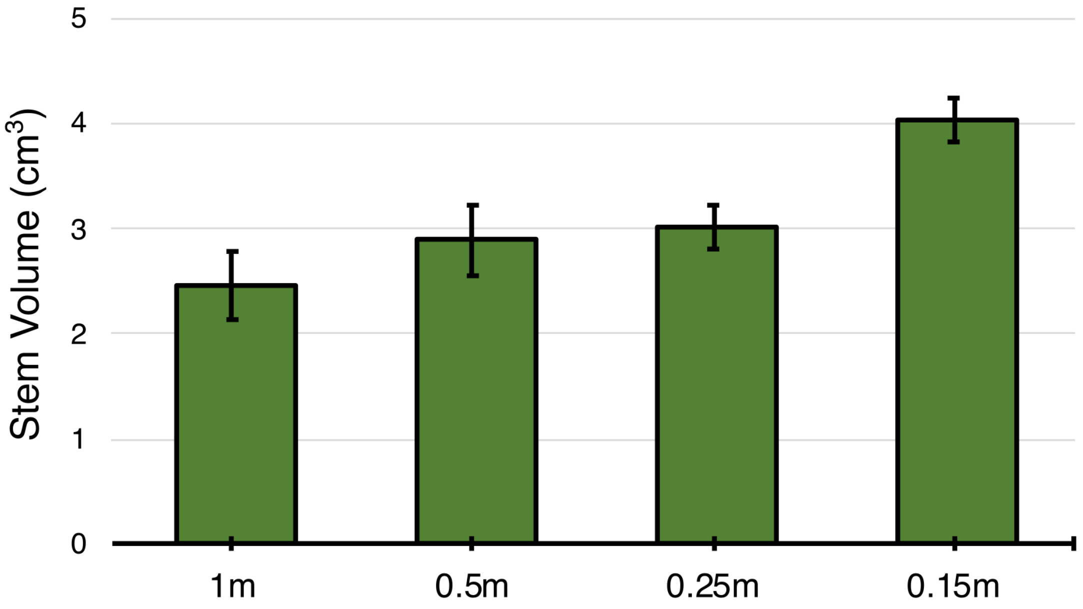

Stem Volume	1 m	±	0.5 m	±	0.25 m	±	0.15 m	±
60DAE	2.45	0.32	2.90	0.34	3.03	0.21	4.03	0.21

Supplementary Table S1: Stem volume of plants grown at 1 m , 0.5 m, 0.25 m and 0.15 m spacing. One-way ANOVA indicated statistically significant differences between densities for stem volume at 60 DAE: [F(3, 16)=4.56, p=0.017]. Tukey post hoc analysis shows statistical variation between 1 m and 0.15 m only .

Leaf Blade Length	1 m	SE	0.5 m	SE	0.25 m	SE	0.15 m	SE
**phy 6**	**81.8**	2.7	**84.2**	4.7	**89.6**	2.8	**88.2**	1.6
**phy 7**	**80.2**	4.5	**84.4**	3.2	**88.6**	2.6	**86.8**	2
**phy 8**	**80**	4.5	**84.2**	4.6	**92.6**	2.2	**87.2**	1.9
**Leaf Blade Width**	**1 m**	SE	**0.5 m**	SE	**0.25 m**	SE	**0.15 m**	SE
**phy 6**	**9.2**	0.2	**9.9**	0.5	**9.2**	0.2	**8.6**	0.2
**phy 7**	**10**	0.3	**9.2**	0.6	**9.2**	0.2	**9**	0.3
**phy 8**	**10.4**	0.2	**9.9**	0.6	**9.1**	0.2	**9.5**	0.3

Supplementary Table S2. Length and width of leaf blades of phytomers 6-8 of plants grown at 1 m, 0.5 m, 0.25 m, and 0.15 m spacing. One-way ANOVA indicated statistically significant differences in leaf blade width of phytomer 6. [$F(3, 16)=1.09$, $p= 0.05$]

Transcript ID	Function	Stem 2mm juvenile	Stem 1cm vegetative	Internode growing floral initiation	Internode growing upper floral initiation	Internode mature floral initiation	Stem mid-internode anthesis	Internode subtending peduncle anthesis	Stem mid-internode grain maturity	Internode subtending peduncle grain maturity	Leaf lower juvenile	Leaf blade juvenile	Leaf upper juvenile
Sobic. 003G045900.1	gibberellin 3-oxidase 2	0	1	0	1	0	1	3	4	2	3	0	1
Sobic. 009G064700.1	gibberellin 3-oxidase 1	0	0	0	0	0	0	0	0	0	0	0	0

Transcript ID	Leaf lower whorl vegetative	Leaf middle whorl vegetative	Leaf upper whorl vegetative	Leaf lower growing floral initiation	Leaf upper growing floral initiation	Leaf lower growing anthesis	Leaf upper growing anthesis	Leaf lower growing grain maturity	Leaf upper growing grain maturity	Leaf sheath growing anthesis	Leaf sheath growing floral initiation	Leaf sheath growing grain maturity	Peduncle floral initiation
Sobic. 003G045900.1	0	3	2	0	0	0	0	0	0	0	0	0	1
Sobic. 009G064700.1	0	0	0	0	0	0	0	0	0	0	0	0	0

Transcript ID	Panicle floral initiation	Panicle lower anthesis	Panicle upper anthesis	Seed dry grain maturity	Seed imbibed grain maturity	Root bottom juvenile	Root top juvenile	Root bottom vegetative	Root top vegetative	Root bottom floral initiation	Root middle floral initiation	Root bottom anthesis	Root bottom grain maturity	FC	FDR
Sobic. 003G045900.1	1	4	1	35	70	1	2	0	4	0	3	0	1	>70	1.8E-11
Sobic. 009G064700.1	0	0	0	0	0	0	0	0	0	0	0	0	0	-	-

Supplementary Table S3: Expression of the GA 3-oxidases. BTx623 tissue and developmental series from McCormick et al. (2018). Data is the average of three biological replicates that are TPM normalized RNA-seq data. Differential expression (FC) and FDR were calculated using EdgeR. Fold change is the difference between the highest and lowest expression values.
